# Variants in human papillomavirus receptor and associated genes are associated with type-specific HPV infection and lesion progression of the cervix

**DOI:** 10.18632/oncotarget.9510

**Published:** 2016-05-20

**Authors:** Jian Zou, Zhu Cao, Jianyang Zhang, Tingting Chen, Shizhou Yang, Yongjie Huang, Die Hong, Yang Li, Xiaojing Chen, Xinyu Wang, Xiaodong Cheng, Weiguo Lu, Xing Xie

**Affiliations:** ^1^ The Department of Gynecologic Oncology, Women's Hospital, School of Medicine, Zhejiang University, Hangzhou, Zhejiang 310006, China; ^2^ Women's Reproductive Health Laboratory of Zhejiang Province, Women's Hospital, School of Medicine, Zhejiang University, Hangzhou, Zhejiang 310006, China

**Keywords:** HPV receptor, SNP, type-specific HPV infection, cervical lesions

## Abstract

Human papillomavirus (HPV) infects cervical epithelial cells through cellular membrane receptors, and then induces the initiation and progression of cervical cancer. Single nucleotide polymorphisms (SNPs) may impact the susceptibility and outcome of diseases, but it's still unknown whether variant in HPV receptor and associated genes is associated with type-specific HPV infection and cervical lesion progression. We examined 96 SNPs in 8 genes which may participate in the HPV infection process in 875 samples with HPV negative or single HPV16, 18, 52, 58 positive from 3299 cervical exfoliated cell samples, by Illumina BeadXpress VeraCode platform, and analyzed the correlation between the SNPs and type-specific HPV infection and cervical lesions progression. We found rs28384376 in *EGFR* and rs12034979 in *HSPG2* significantly correlated to HPV16 infection; rs2575738, rs2575712, rs2575735 in *SDC2* and rs6697265 in *HSPG2* significantly correlated to HPV18 infection; rs10510097 in *FGFR2*, rs12718946 in *EGFR* significantly correlated to HPV52 infection; rs4947972 in *EGFR*, rs2981451 in *FGFR2*, rs2575735 in *SDC2* significantly correlated to HPV58 infection. And rs3135772, rs1047057 and rs2556537 in *FGFR2*, rs12034979 in *HSPG2*, rs16894821 in *SDC2* significantly correlated to cervical lesion progression induced by HPV16 infection; rs6697265 and rs6680566 in *HSPG2*, rs16860426 in *ITGA6* by HPV18 infection; rs878949 in *HSPG2*, rs12718946 and rs12668175 in *EGFR* by HPV52 infection; no SNP by HPV58 infection. Our findings suggest that HPV receptor and associated gene variants may influence the susceptibilities to HPV type-specific infection and cervical lesion progression, which might have a potential application value in cervical cancer screening and therapy.

## INTRODUCTION

Cervical cancer is the second most common cancer among women, with more than 527,624 new cases and 265,672 deaths [1], in the world. Among these, 85% of the cervical cancer cases occur in developing countries [2]. The etiology of cervical cancer has already been identified, that is, persistent infection of high-risk HPV is a causal factor for cervical lesions and cervical cancer [3]. So far, there are 160 genotypes HPV have been found, among those, 13 high-risk types are related to the development of cervical cancer [4]. However, infectious frequency of each high-risk type is not same, and HPV16 and 18 are most common. In the worldwide, the prevalence of HPV16 and 18 in women with normal cytology are 2.8% and 1.1%, while the prevalence of HPV52 and 58 are 1.5% and 1.0%, respectively. Additionally, the prevalence of HPV 52 and 58 in normal cytology in China and East Asia appears some higher, accounting for 2.8% and 1.7%, than that in western areas [1].

The differences of HPV genotype distribution are also found in different grades of cervical lesions. A meta-analysis showed that HPV16 positivity was gradually increased from normal/ASCUS/LSIL/CIN1 (20-28%), through CIN2/HSIL (40/47%) to CIN3/ICC (58/63%)[5]. Another study revealed that HPV16 and HPV18 were 2-fold and 1.5-fold, respectively, more common in SCC than those in HPV-positive LSIL, while HPV52 and 58 were less common in SCC compared with HPV-positive LSIL, with SCC/LSIL ratios of 0.28 to 0.85 [6]. Those different distributions of HPV genotypes represent the different infectious and carcinogenic potentials of HPVs, but it is still unknown whether they are also related to the genetic susceptibility of individual to HPV type-specific infection.

Previous studies have revealed that viruses infect host cells through the relevant receptors, such as herpes virus entry mediator(HVEM) for Herpes virus, *CD4*, as well as *CXCR4 and CCR5*, for HIV [7], and sodium taurocholate cotransporting polypeptide (*NTCP*) for HBV [8]. Further, researches also show that single nucleotide polymorphisms (SNPs) in virus receptors can affect the infection process and outcome of pathogens. For example, a mutation in the chemokine receptor CCR5-Δ32, a co-receptor for macrophage-tropic (M-tropic) HIV-1 strains, can increase the host tolerance and promote the progression of the disease [9], and SNPs in syndecan 2 gene were associated with HIV DNA levels [10]. It has been known that multiple receptor engagements are involved in the process of HPV infecting human cervical epithelial cells [11–16]. According to studies on HPV16, virion binds to heparin sulfate proteoglycans (HSPGs) on either the epithelial cell surface or basement membrane through interactions with the L1 major capsid protein [17–18]. Growth factor receptors become activated through HSPG/growth factor/virion complexes that initiate signaling cascades during early virion-host cell interactions. After binding to HSPGs, virion undergoes conformational changes, leading to isomerization by cyclophilin B and proprotein convertase(*FURIN*) mediated L2 minor capsid protein cleavage that increases L2 N terminus exposure. Along with binding to HSPGs, virion binds to alpha 6 integrins, which initiate further intracellular signaling events. Following these primary binding events, HPV16 binds to a newly identified L2-specific receptor, the annexin A2 heterotetramer. Therefore, as opposed to a sequential handoff of the virion from one receptor to another, we hypothesize that a receptor complex coalesces and includes HSPGs(*HSPG2* and *SDC2*), CyPB(*PPIB*), alpha 6 integrin(*ITGA6*), tetraspanins(*TSPAN1*), GFR(*EGFR and FGFR2*) and A2t. Thus, we selected eight genes, which have been identified as mainly molecules involved in HPV infection process, into our study. The eight genes included *EGFR, PPIB, HSPG2, FGFR2, FURIN, ITGA6, TSPAN1* and *SDC2*). Considering the association of individual SNP with pathogen infection and disease outcome, we assume that SNPs in HPV receptor and associated genes may influence the susceptibility to type-specific HPV infection and cervical lesions progression.

Thus, we selected 96 single-nucleotide polymorphism (SNP) sites in the eight genes that were reported to be involved in the process of HPV infection using the haploview software, and evaluated the correlation between the distribution frequency of various SNP sites and type-specific HPV infection and cervical lesion progression in four common HPV genotypes (HPV16/18/52/58) in China and East Asia. Our study aimed to find out the genetic susceptibility to type-specific HPV infection and cervical lesion progression and search for a novel strategy for cervical cancer prevention or therapy.

## RESULTS

### The age distribution in the samples

The median age of 875 women whose cervical samples were collected was 41 yrs (21-69 yrs), included 40 yrs (22-68 yrs) in 214 controls (HPV negative), 43 yrs(21-69 yrs) in 294 single HPV16 positive, 44 yrs(27-59yrs) in 55 single HPV 18 positive, 40 yrs(21-69 yrs) in 155 single HPV 52 positive, and 41 yrs(23-68 yrs) in 157 single HPV 58 positive. Further, the median age in 502 women with ≤LSIL and 373 with ≥HSIL was 40 yrs(21-63 yrs) and 43 yrs(21-69 yrs), respectively.

### The significant different SNP sites, genotypes and haplotypes between single HPV16/18/52/58 positive and HPV negative in all the samples

All the SNPs in control population of this study were tested by Hardy–Weinberg equilibrium (HWE) as shown in Supplementary Table S1. Further, the differences in frequency distributions of alleles between cases and controls were compared by χ^2^ test and fisher's test.

#### SNP sites

Compared with HPV negative, there were two significant SNPs in *SDC2* gene (rs2651465, p=0.01449, OR: 0.7154, 95%CI: 0.5487-0.9328 and rs2515127, p=0.03553, OR: 1.409, 95%CI: 1.032-1.924) and one in *EGFR* (rs4947972, p=0.02629, OR: 1.544, 95%CI: 1.06-2.25) in HPV16 positive group. There were three significant SNPs in *SDC2* gene (rs2575712, p=0.001118, OR: 0.4829, 95%CI: 0.3101-0.7519; rs2575735, p=0.03549, OR: 1.725, 95%CI: 1.069-2.782 and rs2575738, p=0.04269, OR: 1.639, 95%CI: 1.026-2.62) and two in *HSPG2* gene (rs3767137, p=0.00345, OR: 0.3469, 95%CI: 0.1621-0.7424 and rs6658920, p=0.04755, OR: 0.435, 95%CI: 0.1927-0.9822) and one in *TSPAN1* gene (rs10890384, p=0.03999, OR: 0.4111, 95%CI: 0.1719-0.9831) in HPV18 positive group. There was one significant SNP in *SDC2* gene (rs2589205, p=0.02931, OR: 1.402, 95%CI: 1.044-1.884), one in *HSPG2* gene (rs6680566, p= 0.0302, OR: 0.7112, 95%CI: 0.5229-0.9674) and one in *PPIB* gene (rs2253557, p=0.04421, OR: 0.4602, 95%CI: 0.2208-0.9591) in HPV52 positive group. There were two significant SNPs in *EGFR* gene (rs11770506, p=0.01617, OR: 1.466, 95%CI: 1.082-1.985 and rs4947972, p=0.02831, OR: 1.629, 95%CI: 1.065-2.493) and one in *FURIN* gene (rs17514846, p=0.02622, OR: 0.5896, 95%CI: 0.3725-0.9332) and one in *SDC2* gene (rs2575712, p=0.03558, OR: 0.7214, 95%CI: 0.537-0.9692) in HPV58 positive group. The detailed data were shown in Supplementary Table S1.

#### Genotypes

Compared with HPV negative, there was one protective SNP genotype “TA” of rs2651465 (OR: 0.624314, 95%CI: 0.419429-0.926093, p=0.016993) in HPV16 positive group. Three protective SNP genotypes “AA” and “AC” of r s2575712 (OR: 0.272678, 95%CI: 0.096587-0.698705, p=0.004221 and OR: 0.393682, 95%CI: 0.187654-0.818321, p=0.00791) and “AG” of r s3767137 (OR: 0.271899, 95%CI: 0.090299-0.682749, p=0.002961) in HPV18 positive group. There was one susceptible SNP genotype “AA” of rs2589205 (OR: 2.269532, 95%CI: 1.114481-4.681472, p=0.019098) and one protective SNP genotype “GA” of rs6680566 (OR: 0.54901, 95%CI: 0.339422-0.884265, p=0.011555) in HPV52 positive group. There were two susceptible SNP genotypes “AG” of rs11770506 (OR: 2.100947, 95%CI: 1.297139-3.429275, p=0.001953) and “GC” of rs4947972 (OR: 1.700056, 95%CI: 1.029087-2.814845, p=0.028967), two protective SNP genotypes “AC” of rs17514846 (OR: 0.517462, 95%CI: 0.29487-0.889611, p=0.012366) and “AA” of rs2575712 (OR: 0.476464; 95%CI: 0.23888-0.935745, p=0.02684) in HPV58 positive group. The detailed data were shown in Supplementary Table S2.

#### Haplotypes

Compared with HPV negative, there was no significant different haplotype in HPV16 positive group. There is one protective haplotype “GGAGA” (rs7518070, rs4654771, rs3767137, rs12117402, rs2305562) in *HSPG2* gene (Freq (case) =0.057, Freq (control) =0.16, χ^2^=7.612, Chi square's P value=0.0058, Fisher's P value=0.004508) in HPV18 positive group. There were one protective haplotype “GGG”(rs2254357, rs878949, rs6680566) in *HSPG2* (Freq(case)=0.326, Freq (control) =0.403, χ^2^=4.421, Chi square's P value=0.0355, Fisher's P value =0.036331) and one susceptible haplotype “GGA” (rs2254357, rs878949, rs6680566) in *HSPG2* (Freq (case) =0.295, Freq (control)= 0.228, χ^2^=4.305, Chi square's P value =0.038, Fisher's P value=0.039358) in HPV52 positive group. There were two protective haplotypes “GG” (rs11770506, rs763317) in *EGFR* (Freq (case) =0.578, Freq (control)=0.667, χ^2^=6.133, Chi square's P value=0.0133, Fisher's P value =0.01617) and “AA” (rs17514846, rs4702) in *FURIN* (Freq (case)=0.097, Freq (control)=0.152, χ^2^=4.724, Chi square's P=0.0297, Fisher's P value=0.043411) in HPV58 positive group. The detailed data were shown in Supplementary Table S3.

### The significant different SNP sites, genotypes and haplotypes between single HPV16/18/52/58 positive and HPV negative in the normal samples

All of the tested SNPs in the table were in Hardy–Weinberg equilibrium (HWE) in the control population from the normal samples (p>0.05), as shown in Table 1A. The different genotypes of individual SNPs in each analyzed genes in HPV negative samples (214 cases) were shown in Supplementary Table S4.

**Table 1A:** The significant different SNP sites in target genes between single HPV16/18/52/58 positive and HPV negative in the normal samples

VS HPV negative	SNP_ID	Gene	chr	position	A1	A2	HWE p value in control	OR(95%CI)	P value
**HPV16**	**rs28384376**	***EGFR***	**7**	**55233121**	**A**	**C**	**1**	**2.642 (1.267-5.509)**	**0.01865**
	**rs12034979**	***HSPG2***	**1**	**22259146**	**A**	**G**	**1**	**3.114 (1.209-8.021)**	**0.02327**
**HPV18**	**rs2575738**	***SDC2***	**8**	**97530402**	**A**	**G**	**1**	**2.128 (1.108-4.086)**	**0.02623**
	**rs6697265**	***HSPG2***	**1**	**22256725**	**G**	**C**	**0.4326**	**1.978 (1.059-3.695)**	**0.03901**
	**rs2575712**	***SDC2***	**8**	**97576436**	**A**	**C**	**0.8779**	**0.4909 (0.258-0.934)**	**0.04014**
	**rs2575735**	***SDC2***	**8**	**97534651**	**A**	**G**	**0.6067**	**2.056 (1.034-4.088)**	**0.04542**
**HPV52**	**rs10510097**	***FGFR2***	**10**	**123327876**	**A**	**G**	**1**	**1.711 (1.081-2.709)**	**0.02673**
	**rs12718946**	***EGFR***	**7**	**55221447**	**G**	**C**	**1**	**0.6108 (0.3986-0.9357)**	**0.02796**
**HPV58**	**rs4947972**	***EGFR***	**7**	**55161043**	**G**	**C**	**1**	**1.977 (1.088-3.59)**	**0.02882**
	**rs2981451**	***FGFR2***	**10**	**123278914**	**A**	**C**	**1**	**0.5318 (0.3039-0.9306)**	**0.02921**
	**rs2575735**	***SDC2***	**8**	**97534651**	**A**	**G**	**0.6067**	**1.77 (1.063-2.947)**	**0.03599**

A1: Minor allele name

A2: Major allele name

OR: Estimated odds ratio (for A1, A2 is reference)

HWE: Hardy–Weinberg equilibrium

#### SNP sites

Compared with HPV negative in the normal samples, there was one significant SNP in *EGFR* gene (rs28384376, p=0.01865, OR: 2.642, 95%CI: 1.267-5.509) and one in *HSPG2* gene (rs12034979, p=0.02327, OR: 3.114, 95%CI: 1.209-8.021) in HPV16 positive group. There were three SNPs in *SDC2* gene (rs2575738, p=0.02623, OR: 2.128, 95%CI: 1.108-4.086; rs2575712, p=0.04014, OR: 0.4909, 95%CI: 0.258-0.934 and rs2575735, p=0.04542, OR: 2.056, 95%CI: 1.034-4.088) and one in *HSPG2* gene (rs6697265, p=0.03901, OR: 1.978, 95%CI: 1.059-3.695) in HPV18 positive group. There was one SNP in *FGFR2* gene (rs10510097, p=0.02673, OR: 1.711, 95%CI: 1.081-2.709) and one in *EGFR* gene (rs12718946, p= 0.02796, OR: 0.6108, 95%CI: 0.3986-0.9357) in HPV52 positive group. There was one SNP in *EGFR* gene (rs4947972, p=0.02882, OR: 1.977, 95%CI: 1.088-3.59) and one in *FGFR2* gene (rs2981451, p=0.02921, OR: 0.5318, 95%CI: 0.3039-0.9306) and one in *SDC2* gene (rs2575735, p=0.03599, OR: 1.77, 95%CI: 1.063-2.947) in HPV58 positive group. The detailed data were shown in Table 1A.

#### Genotypes

Compared with HPV negative in the normal samples, there were two susceptible SNP genotypes “AA” of 28384376 (OR: 21.32744, 95%CI: 1.621665-1158.865, p=0.007919) and “AG” of rs12034979 (OR: 3.437163; 95%CI: 1.059898-10.29703, p= 0.019656) in HPV16 positive group. Three susceptible SNP genotypes “AA” of rs2575738 (OR: 5.092509, 95%CI: 0.954255-23.63242, p=0.028565), “GG” of rs6697265 (OR: 5.981678, 95%CI: 0.986387-64.47637, p=0.047442) and “GC” of rs6697265 (OR: 4.600737, 95%CI: 1.008827-42.96709, p=0.0334) in HPV18 positive group. There was one protective genotype “GG” of rs12718946 (OR: 0.147506, 95%CI: 0.015969-0.652735, p=0.00389) in HPV52 positive group. There was one protective genotype “AA” of rs2981451 (OR: 0, 95%CI: 0-0.894449, p=0.038732) in HPV58 positive group (Table 1B).

**Table 1B:** The significant different genotypes in target genes between single HPV16/18/52/58 positive and HPV negative in the normal samples

VS HPV negative	SNP Number/Gene	genotype	No.(frequency) in case	No.(frequency) in control	OR(95%CI)	P fisher
**HPV16**	**rs28384376** ***EGFR***	**AA** **AC** **CC**	**3(10.7%)** **6(21.4%)** **19(67.9%)**	**1(0.6%)** **30(17.5%)** **140(81.9%)**	**21.32744 (1.621665-1158.865)** **1.470486 (0.442615-4.260336)** **ref**	**0.007919** **0.418286** **ref**
	**rs12034979** ***HSPG2***	**AA** **AG** **GG**	**0(0.0%)** **7(25.0%)** **21(75.0%)**	**0(0.0%)** **15(8.8%)** **156(91.2%)**	**0(0-INF)** **3.437163 (1.059898-10.29703)** **ref**	**1** **0.019656** **ref**
**HPV18**	**rs2575738** ***SDC2***	**AA** **AG** **GG**	**4(17.4%)** **9(39.1%)** **10(43.5%)**	**8(4.7%)** **57(33.7%)** **104(61.6%)**	**5.092509(0.954255-23.63242)** **1.637461(0.553659-4.778623)** **ref**	**0.028565** **0.323045** **ref**
	**rs6697265** ***HSPG2***	**GG** **GC** **CC**	**6(26.1%)** **15(65.2%)** **2(8.7%)**	**27(15.8%)** **89(52.0%)** **55(32.2%)**	**5.981678(0.986387-64.47637)** **4.600737(1.008827-42.96709)** **ref**	**0.047442** **0.0334** **ref**
	**rs2575712** ***SDC2***	**AA** **AC** **CC**	**3(13.0%)** **10(43.5%)** **10(43.5%)**	**45(26.6%)** **86(50.9%)** **38(22.5%)**	**0.256792(0.042376-1.093154)** **0.444594(0.151795-1.299012)** **ref**	**0.070437** **0.123706** **ref**
	**rs2575735** ***SDC2***	**AA** **AG** **GG**	**3(13.0%)** **8(34.8%)** **12(52.2%)**	**6(3.5%)** **48(28.1%)** **117(68.4%)**	**4.784701(0.68814-26.16497)** **1.620441(0.538477-4.632181)** **ref**	**0.058901** **0.314344** **ref**
**HPV52**	**rs10510097** ***FGFR2***	**AA** **AG** **GG**	**6(8.7%)** **27(39.1%)** **36(52.2%)**	**6(3.5%)** **52(30.4%)** **113(66.1%)**	**3.112224(0.779492-12.44474)** **1.626255(0.854198-3.082385)** **ref**	**0.080975** **0.120916** **ref**
	**rs12718946** ***EGFR***	**GG** **GC** **CC**	**2(2.9%)** **36(52.2%)** **31(44.9%)**	**27(15.8%)** **83(48.5%)** **61(35.7%)**	**0.147506(0.015969-0.652735)** **0.854126(0.457694-1.597197)** **ref**	**0.00389** **0.655333** **ref**
**HPV58**	**rs4947972** ***EGFR***	**GG** **GC** **CC**	**2(3.8%)** **16(30.2%)** **35(66.0%)**	**1(0.6%)** **34(19.9%)** **136(79.5%)**	**7.646792(0.387837-460.2661)** **1.823182(0.840407-3.86191)** **ref**	**0.11474** **0.12553** **ref**
	**rs2981451** ***FGFR2***	**AA** **AC** **CC**	**0(0.0%)** **18(34.0%)** **35(66.0%)**	**13(7.6%)** **69(40.4%)** **89(52.0%)**	**0(0-0.894449)** **0.664629(0.324939-1.325018)** **ref**	**0.038732** **0.259641** **ref**
	**rs2575735** ***SDC2***	**AA** **AG** **GG**	**4(7.6%)** **21(39.6%)** **28(52.8%)**	**6(3.5%)** **48(28.1%)** **117(68.4%)**	**2.762719(0.53651-12.57202)** **1.822717(0.890965-3.700628)** **ref**	**0.216886** **0.082503** **ref**

#### Haplotypes

Compared with HPV negative in the normal samples, there were two critically susceptible haplotypes “GGGG” (Freq(case)= 0.09, Freq (control)= 0.032, χ^2^=4.149, Chi square's P value =0.0417, Fisher's P value =0.059048) and “GGAA” (Freq(case)= 0.037, Freq (control)= 0.006, χ^2^=4.395, Chi square's P value =0.0361, Fisher's P value =0.096763) in block 10 (rs2912780, rs2981578, rs2981575, rs2936870) in *FGFR2* gene and one critically protective haplotype “GA” (Freq(case)= 0.161, Freq (control)= 0.287, χ^2^=3.876, Chi square's P value =0.049, Fisher's P value =0.051813) in block 7 (rs2464474, rs16894821) in *SDC2* gene in HPV16 positive group. There was one susceptible haplotype “GGG” in block3 (rs4654773, rs6697265, rs6658920) (Freq(case)= 0.587, Freq (control)= 0.414, χ^2^=4.93, Chi square's P value =0.0264, Fisher's P value =0.0386) and two protective haplotypes “GGA” in block2 (rs2254357, rs878949, rs6680566) (Freq(case)= 0.108, Freq (control)= 0.243, χ^2^=4.209, Chi square's P value =0.0402, Fisher's P value =0.040537) and “GGAGA” in block1 (rs7518070, rs4654771, rs3767137, rs12117402, rs2305562) (Freq(case)= 0.043, Freq (control)= 0.157, χ^2^=4.26, Chi square's P value =0.039, Fisher's P value =0.042397) in *HSPG2* gene in HPV18 positive group. There were two susceptible haplotypes “GGGAA” (Freq(case)= 0.225, Freq (control)= 0.148, χ^2^=4.164, Chi square's P value =0.0413, Fisher's P value =0.043573) in block 1 (rs7518070, rs4654771, rs3767137, rs12117402, rs2305562) and “GGA” (Freq(case)= 0.333, Freq (control)= 0.244, χ^2^=3.958, Chi square's P value =0.0466, Fisher's P value =0.05283) in block2 (rs2254357, rs878949, rs6680566) in *HSPG2* gene in HPV52 positive group. There was one susceptible haplotype “GC” (Freq(case)= 0.311, Freq (control)= 0.184, χ^2^=7.765, Chi square's P value =0.0053, Fisher's P value =0.006766) and one protective haplotype “GA” (Freq(case)= 0.17, Freq (control)= 0.278, χ^2^=5.001, Chi square's P value =0.0253, Fisher's P value =0.029213) in block 8 (rs3135761, rs2981451) in *FGFR2* gene in HPV58 positive group (Table 1C).

**Table 1C:** The significant different haplotypes in target genes between single HPV16/18/52/58 positive and HPV negative in the normal samples

vs HPV negative	Block	GENE	Haplotype	Freq (case)	Freq (control)	χ^2^	P. Chi Square	OR(95%CI)	P fisher
**HPV16**	**10**	***FGFR2***	**GGAA**	**0.037**	**0.006**	**4.395**	**0.0361**	**6.248476 (0.444392-88.06148)**	**0.096763**
	**10**	***FGFR2***	**GGGG**	**0.09**	**0.032**	**4.149**	**0.0417**	**2.939017 (0.768282-9.649561)**	**0.059048**
	**7**	***SDC2***	**GA**	**0.161**	**0.287**	**3.876**	**0.049**	**0.477545 (0.198019-1.034453)**	**0.051813**
**HPV18**	**3**	***HSPG2***	**GGG**	**0.587**	**0.414**	**4.93**	**0.0264**	**1.997866 (1.025384-3.962219)**	**0.0386**
	**2**	***HSPG2***	**GGA**	**0.108**	**0.243**	**4.209**	**0.0402**	**0.381305 (0.113855-1.009317)**	**0.040537**
	**1**	***HSPG2***	**GGAGA**	**0.043**	**0.157**	**4.26**	**0.039**	**0.243004 (0.027721-0.98132)**	**0.042397**
**HPV52**	**1**	***HSPG2***	**GGGAA**	**0.225**	**0.148**	**4.164**	**0.0413**	**1.689901 (0.987842-2.860856)**	**0.043573**
	**2**	***HSPG2***	**GGA**	**0.333**	**0.244**	**3.958**	**0.0466**	**1.558764 (0.986012-2.450724)**	**0.05283**
**HPV58**	**8**	***FGFR2***	**GC**	**0.311**	**0.184**	**7.765**	**0.0053**	**1.998545 (1.177214-3.360967)**	**0.006766**
	**8**	***FGFR2***	**GA**	**0.17**	**0.278**	**5.001**	**0.0253**	**0.532508 (0.285894-0.949775)**	**0.029213**

Taken above results together, some variants in HPV receptor and associated genes were found to be correlated to type-specific HPV infection, including *EGFR* and *HSPG2* gene to HPV16 infection, *SDC2* and *HSPG2* to HPV18 infection, *EGFR*, *FGFR2* and *HSPG2* to HPV52 infection, and *EGFR*, *FGFR2* and *SDC2* to HPV58 infection

### The significant different SNP sites, genotypes and haplotypes between ≥HSIL and ≤LSIL in single HPV16/18/52/58 positive subgroups

All of the tested SNPs in the table were in Hardy–Weinberg equilibrium (HWE) in the control population of this study (p>0.05 except for rs878949 and rs12668175 in HPV52), as shown in Table 2A.

**Table 2A:** The significant different SNP sites between ≥ HSIL and ≤ LSIL in single HPV16/18/52/58 positive subgroups

Pathologic degrees	VS ≤LSIL	SNP_ID	Gene	chr	position	A1	A2	HWE p value in control	OR (95%CI)	P value
**HPV16**	**≥HSIL**	**rs3135772**	***FGFR2***	**10**	**123263616**	**A**	**G**	**1**	**0.5175 (0.3305-0.8104)**	**0.004776**
		**rs2556537**	***FGFR2***	**10**	**123241794**	**G**	**A**	**0.7489**	**1.863 (1.165-2.978)**	**0.009159**
		**rs12034979**	***HSPG2***	**1**	**22259146**	**A**	**G**	**1**	**0.3534 (0.1577-0.792)**	**0.01753**
		**rs1047057**	***FGFR2***	**10**	**123239112**	**A**	**G**	**0.7456**	**1.694 (1.067-2.689)**	**0.03118**
		**rs16894821**	***SDC2***	**8**	**97604112**	**G**	**A**	**0.3204**	**0.5824 (0.3598-0.9429)**	**0.03392**
**HPV18**	**≥HSIL**	**rs6697265**	***HSPG2***	**1**	**22256725**	**C**	**G**	**0.4756**	**2.923 (1.245-6.865)**	**0.0213**
		**rs6680566**	***HSPG2***	**1**	**22229090**	**G**	**A**	**0.4732**	**0.3646 (0.1538-0.8643)**	**0.02301**
		**rs16860426**	***ITGA6***	**2**	**173319112**	**A**	**T**	**0.714**	**0.3766 (0.1433-0.9895)**	**0.04799**
**HPV52**	**≥HSIL**	**rs878949**	***HSPG2***	**1**	**22227091**	**A**	**G**	**0.01918**	**2.479 (1.287-4.776)**	**0.00897**
		**rs12718946**	***EGFR***	**7**	**55221447**	**G**	**C**	**0.4186**	**1.887 (1.096-3.248)**	**0.02555**
		**rs12668175**	***EGFR***	**7**	**55178579**	**C**	**A**	**0.02953**	**1.858 (1.073-3.215)**	**0.03583**

A1: Minor allele name

A2: Major allele name

OR: Estimated odds ratio (for A1, A2 is reference)

HWE: Hardy–Weinberg equilibrium

#### SNP sites

Compared to single HPV16 positive with ≤LSIL, there were three significant different SNPs in *FGFR2* gene (rs3135772, p=0.004776, OR: 0.5175, 95%CI: 0.3305-0.8104; rs2556537, OR: 1.863, p=0.009159, 95%CI: 1.165-2.978 and rs1047057, p=0.03118, OR: 1.694, 95%CI: 1.067-2.689) and one (rs12034979, p=0.01753, OR: 0.3534, 95%CI: 0.1577-0.792) in *HSPG2* gene and one (rs16894821, p=0.03392, OR: 0.5824, 95%CI: 0.3598-0.9429) in *SDC2* gene in single HPV16 positive with ≥HSIL. There were two significant different SNPs in *HSPG2* gene (rs6697265, p=0.0213, OR: 2.923, 95%CI: 1.245-6.865 and rs6680566, p=0.02301, OR: 0.3646, 95%CI: 0.1538-0.8643) and one in *ITGA6* gene (rs16860426, p=0.04799, OR: 0.3766, 95%CI: 0.1433-0.9895) in HPV18 positive with ≥HSIL. There were two significant different SNPs in *EGFR* gene (rs12718946, p=0.02555, OR: 1.887, 95%CI: 1.096-3.248; and rs12668175, p=0.03583, OR: 1.858, 95%CI: 1.073-3.215) and one in *HSPG2* gene (rs878949, p=0.00897, OR: 2.479, 95%CI: 1.287-4.776) in HPV52 positive with ≥HSIL. There was no significant different SNP site in HPV58 positive with ≥HSIL (Table 2A).

#### Genotypes

Compared to single HPV16 positive with ≤LSIL, there were four protective SNP genotypes “AA” (OR: 0.26792, 95%CI: 0.087236-0.740245, p=0.006378) and “AG” (OR: 0.404562, 95%CI:0.139783-1.031401, p=0.047019) of rs3135772; “AG” (OR:0.2899; 95%CI: 0.113434-0.771219, p=0.006208) of rs12034979 and “GA” (OR:0.488314, 95%CI: 0.240851-0.978606, p=0.031276) of rs16894821; two susceptible SNP genotypes “GG” (OR: 3.781242, 95%CI: 1.167225-16.15924, p=0.021072) of rs2556537 and “AG” (OR:2.263455, 95%CI:1.087514-4.74233, p=0.02074) of rs1047057 in single HPV16 positive with ≥HSIL. There was a susceptible SNP genotype “CC” (OR: 8.913675, 95%CI: 1.11492-123.4335, p=0.036075) of rs6697265 and two protective SNP genotypes “GG” (OR: 0.152201, 95%CI: 0.011374-1.184621, p=0.04718) and “GA” (OR:0.21407, 95%CI: 0.040022-1.000252, p= 0.040811) of rs6680566 in HPV18 positive with ≥HSIL. There were two susceptible SNP genotypes “AG” (OR:3.771844, 95%CI: 1.490165-9.6238, p=0.002348) of rs878949 and “GG” (OR:4.101835, 95%CI: 1.034313-17.37973, p=0.024721) of rs12718946 in HPV52 positive with ≥HSIL. There was no significant different SNP genotype in HPV58 positive with ≥HSIL (Table 2B).

**Table 2B:** The significant different genotypes between ≥HSIL and ≤LSIL in single HPV16/18/52/58 positive subgroups

Pathologic degrees	VS ≤LSIL	SNP Number/Gene	genotype	No.(frequency) in case	No.(frequency) in control	OR (95%CI)	P fisher
**HPV16**	**≥HSIL**	**rs3135772** ***FGFR2***	**AA** **AG** **GG**	**49(21.3%)** **105(45.7%)** **76(33.0%)**	**17(35.4%)** **24(50.0%)** **7(14.6%)**	**0.26792 (0.087236-0.740245)** **0.404562 (0.139783-1.031401)** **ref**	**0.006378** **0.047019** **ref**
		**rs2556537** ***FGFR2***	**GG** **GA** **AA**	**43(18.8%)** **124(54.1%)** **62(27.1%)**	**4(8.3%)** **22(45.8%)** **22(45.8%)**	**3.781242(1.167225-16.15924)** **1.993674(0.970782-4.102563)** **ref**	**0.021072** **0.054603** **ref**
		**rs12034979** ***HSPG2***	**AA** **AG** **GG**	**1(0.5%)** **16(7.0%)** **211(92.5%)**	**0(0.0%)** **10(20.8%)** **38(79.2%)**	**Inf(0.004612- Inf)** **0.2899(0.113434-0.771219)** **ref**	**1** **0.006208** **ref**
		**rs1047057** ***FGFR2***	**AA** **AG** **GG**	**42(18.3%)** **126(55.0%)** **61(22.7%)**	**6(12.5%)** **20(41.7%)** **22(45.8%)**	**2.508327(0.887159-8.221692)** **2.263455(1.087514-4.74233)** **ref**	**0.077024** **0.02074** **ref**
		**rs16894821** ***SDC2***	**GG** **GA** **AA**	**8(3.5%)** **84(36.5%)** **138(60.0%)**	**3(6.2%)** **25(52.1%)** **20(41.7%)**	**0.389393(0.084063-2.464575)** **0.488314(0.240851-0.978606)** **ref**	**0.174535** **0.031276** **ref**
**HPV18**	**≥HSIL**	**rs6697265** ***HSPG2***	**CC** **CG** **GG**	**8(44.4%)** **8(44.4%)** **2(11.1%)**	**4(12.5%)** **18(56.3%)** **10(31.2%)**	**8.913675(1.11492-123.4335)** **2.179284(0.335237-25.00107)** **ref**	**0.036075** **0.4528** **ref**
		**rs6680566** ***HSPG2***	**GG** **GA** **AA**	**2(11.1%)** **7(38.9%)** **9(50.0%)**	**8(25.0%)** **19(59.4%)** **5(15.6%)**	**0.152201(0.011374-1.184621)** **0.21407(0.040022-1.000252)** **ref**	**0.04718** **0.040811** **ref**
		**rs16860426** ***ITGA6***	**AA** **AT** **TT**	**1(5.6%)** **5(27.8%)** **12(66.7%)**	**4(12.5%)** **17(53.1%)** **11(34.4%)**	**0.240602(0.004308-2.938929)** **0.277996(0.058925-1.142953)** **ref**	**0.333333** **0.065387** **ref**
**HPV52**	**≥HSIL**	**rs878949** ***HSPG2***	**AA** **AG** **GG**	**2(5.7%)** **15(42.9%)** **18(51.4%)**	**5(4.5%)** **19(17.1%)** **87(78.4%)**	**1.919933(0.170181-12.9159)** **3.771844(1.490165-9.6238)** **ref**	**0.606343** **0.002348** **ref**
		**rs12718946** ***EGFR***	**GG** **GC** **CC**	**8(22.9%)** **21(60.0%)** **6(17.1%)**	**13(11.8%)** **56(50.9%)** **41(37.3%)**	**4.101835(1.034313-17.37973)** **2.544463(0.889958-8.410202)** **ref**	**0.024721** **0.073346** **ref**
		**rs12668175** ***EGFR***	**CC** **CA** **AA**	**12(35.3%)** **14(41.2%)** **8(23.5%)**	**24(21.6%)** **42(37.8%)** **45(40.5%)**	**2.778045(0.90185-9.022859)** **1.864266(0.651283-5.687745)** **ref**	**0.068632** **0.237231** **ref**

#### Haplotypes

Compared to single HPV16 positive with ≤LSIL, there was one protective haplotype “GG” (Freq(case)= 0.209, Freq (control)= 0.319, χ^2^=5.442, Chi square's P value =0.0197, Fisher's P value =0.022419) in block 6 (rs2464474, rs16894821) in *SDC2* gene in HPV16 positive with ≥HSIL. There was one protective haplotype “GG” (Freq(case)= 0.333, Freq (control)= 0.594, χ^2^=6.25, Chi square's P value =0.0124, Fisher's P value =0.021295) in block2 (rs4654773, rs6697265) in *HSPG2* gene in HPV18 positive with ≥HSIL. There was one susceptible haplotype “CAA” (Freq(case)= 0.271, Freq (control)= 0.131, χ^2^=7.681, Chi square's P value =0.0056, Fisher's P value =0.00897) in block2 (rs2254357, rs878949, rs6680566) in *HSPG2* gene in HPV52 positive with ≥HSIL. There was no significant different haplotype in HPV58 positive with ≥HSIL (Table 2C).

**Table 2C:** The significant different haplotypes between ≥HSIL and ≤LSIL in single HPV16/18/52/58 positive subgroups

Hpv genotype	VS≤LSIL	Block	GENE	Haplotype	Freq (case)	Freq (control)	χ^2^	P. Chi Square	OR (95%CI)	P fisher
**HPV16**	**≥HSIL**	**6**	***SDC2***	**GG**	**0.209**	**0.319**	**5.442**	**0.0197**	**0.553624 (0.333747-0.931329)**	**0.022419**
**HPV18**	**≥HSIL**	**2**	***HSPG2***	**GG**	**0.333**	**0.594**	**6.25**	**0.0124**	**0.345927 (0.132108-0.867713)**	**0.021295**
**HPV52**	**≥HSIL**	**2**	***HSPG2***	**CAA**	**0.271**	**0.131**	**7.681**	**0.0056**	**2.470592 (1.20591-4.988233)**	**0.00897**

Taken above results together, some variants in HPV receptor and associated genes were found to be associated with cervical lesion progression induced by type-specific HPV, including *FGFR2*, *HSPG2* and *SDC2* with HPV16, *ITGA6* and *HSPG2* with HPV18, and *EGFR* and *HSPG2* with HPV52, but no with HPV58.

## DISCUSSION

It is well known that the occurrence and development of human cervical cancer are related to high-risk HPV infection. HPV 16 binds to heparin sulfate proteoglycans (HSPGs) on either the epithelial cell surface or basement membrane through interactions with its L1major capsid protein, and afterwards HSPG/growth factor/HPV16 complexes activate growth factor receptors which initiate signaling cascades during early virion-host cell interactions [13]. After HPV enters the cell nucleus, E2 ruptures during the virus gene duplication, which helps the virus to integrate into the host cell chromosome, and consequently the over-expression of E6/E7 gene leads to the development of cervical cancer [19–21]. Although the mechanisms by which HPV induces cervical carcinogenesis have been described already, it is still unknown that the relationship between the variants in HPV receptor and associated genes and the susceptibility to type-specific HPV infection and cervical lesions progression. A haplotype-based association analysis is an increasingly accepted approach for genetic association studies [22], thus we firstly performed a haplotype-based study to analyze the relationship between HPV receptor and associated gene variants and the susceptibility to type-specific HPV infection and cervical lesion progression in Chinese women.

In an analysis on the susceptibility of HPV receptor and associated gene variants to type-specific HPV infection, we found two susceptible SNP sites rs28384376 in *EGFR* gene and rs12034979 in *HSPG2* gene, and two susceptible genotypes “AA” of rs28384376 and “AG” of rs12034979, and two risk haplotypes “GGAA” and “GGGG” in block10 (rs2912780, rs2981578, rs2981575, rs2936870) in *FGFR2* gene and one protective haplotype “GA” in block7 (rs2464474, rs16894821) in *SDC2* gene in HPV16 positive group, suggesting the genetic variants in *EGFR*, *HSPG2*, *FGFR2* and *SDC2* genes may be associated with type-specific HPV16 infection. The differences of haplotype frequency distributions in target genes between cases and controls were significant by χ^2^ test (p<0.05) while not by fisher's test (p>0.05), suggesting that each kind of haplotype may be susceptible to type-specific HPV16 infection and there is no dominant susceptible or protective haplotype. The OR of rs28384376 and rs12034979 are 2.642 and 3.114 respectively, and there was no protective SNP site except for one critical haplotype. All those phenomenons might be employed as an explanation that HPV16 possesses the highest prevalence among all high-risk genotypes in the worldwide. Again we found three susceptible SNP sites rs2575738 and rs2575735 in *SDC2* gene and rs6697265 in *HSPG2* gene, and one protective SNP site rs2575712 in *SDC2* gene, and three susceptible genotypes “AA” of rs2575738 and “GG”, “GC” of rs6697265, and one susceptible haplotype “GGG” in block3 (rs4654773, rs6697265, rs6658920) and two protective haplotypes “GGA” in block2 (rs2254357, rs878949, rs6680566), “GGAGA” in block1 (rs7518070, rs4654771, rs3767137, rs12117402, rs2305562) in *HSPG2* gene in HPV18 positive group, suggesting that genetic variants in *HSPG2* and *SDC2* genes are associated with type-specific HPV18 infection. Similarly, there was one susceptible SNP site rs10510097 in *FGFR2* gene and one protective SNP site rs12718946 in EGFR gene, and one protective SNP genotype “GG” of rs12718946; and two susceptible haplotypes “GGA” in block2 (rs2254357, rs878949, rs6680566), “GGGAA” in block1 (rs7518070, rs4654771, rs3767137, rs12117402, rs2305562) in *HSPG2* gene in HPV52 positive group, suggesting that genetic variants in *HSPG2*, *FGFR2* and *EGFR* genes are associated with type-specific HPV52 infection. And again, there was two susceptible SNP sites rs4947972 in *EGFR* gene and rs2575735 in *SDC2* gene and one protective SNP site rs2981451 in *FGFR2* gene, and one protective SNP genotype “AA” of rs2981451, and one susceptible haplotype “GC” and one protective haplotype “GA” in block8 (rs3135761, rs2981451) in *FGFR2* gene in HPV58 positive group, suggesting that genetic variants in *EGFR*, *SDC2*, and *FGFR2* are associated with type-specific HPV58 infection. Thus, variants of HPV receptor and associated genes in SNP sites, genotypes and haplotypes may influence significantly the susceptibility of the individual to type-specific HPV18, 52 and 58 in Chinese women, but seems unobvious to type-specific HPV16 infection.

Furthermore, we analyze the correlation between HPV receptor and associated gene variants and cervical lesion progression in each single HPV positive group. In HPV16 positive group, there were two susceptible SNP sites rs2556537 and rs1047057 in *FGFR2* gene, and three protective SNP sites rs3135772 in *FGFR2* gene, rs12034979 in *HSPG2* gene and rs16894821 in *SDC2* gene, and two susceptible SNP genotypes “GG” of rs2556537 and “AG” of rs1047057 and four protective SNP genotypes “AA”, “AG” of rs3135772, “AG” of rs12034979, “GA” of rs16894821, and one protective haplotype “GG” in block 6 (rs2464474, rs16894821) in *SDC2* gene, suggesting that genetic variants of *FGFR2*, *HSPG2*, and *SDC2* are related to cervical lesion progression induced by HPV16 infection. In HPV18 positive group, there was one susceptible SNP site rs6697265 in *HSPG2* gene and two protective SNP sites rs6680566 in *HSPG2* gene and rs16860426 in *ITGA6* gene, and one susceptible SNP genotype “CC” of rs6697265 and two protective SNP genotypes “GG” and “GA” of rs6680566, and one protective haplotype “GG” in block2 (rs4654773, rs6697265), suggesting that genetic variants in *HSPG2* and *ITGA6* are related to cervical lesion progression induced by HPV18 infection. In HPV52 positive group, there were three susceptible SNP sites rs878949 in *HSPG2* gene, rs12718946 and rs12668175 in *EGFR* gene, and two susceptible SNP genotype “AG” of rs878949 and “GG” of rs12718946, and one susceptible haplotype “CAA” in block2 (rs2254357, rs878949, rs6680566), suggesting that genetic variants in *HSPG2* and *EGFR* are related to cervical lesion progression induced by HPV52 infection. In HPV58 positive group, there was no significant different genetic variant related to cervical lesion progression. Thus, HPV receptor and associated gene variants may be associated with cervical lesion progression induced by HPV16, 18, and 52, but not HPV58, infection in Chinese women.

## MATERIALS AND METHODS

### Sample collection

Totally 3299 residual cervical exfoliated cell samples were primarily collected from women with various gynecologic disorders who underwent primary HPV testing, HC2 or Cervista test, in Clinic of Gynecologic Oncology of Women's Hospital, Medical School, Zhejiang University from October 2012 to May 2015. Of HPV positive samples, those were excluded just as follows: 1) other than A7/9 positive tested by Cervista, 2) previous surgical or physical therapy to cervix, 3) cervical infection by other pathogens, such as HIV, Syphilis, and Candida, 4) incorporative immune disease or use of immunosuppressive agents, 5) incorporative other malignant tumors, 6) incorporative pregnancy. HPV genotyping was performed for the remaining HPV positive samples using HybriBio's proprietary flow-through hybridization technique, and the single HPV16, 18, 52, 58 positive samples were selected for study. All HPV negative samples were identified by no bands on agarose gel electrophoresis after PCR amplification using HPV L1 consensus PCR primers (primers MY09 and MY11). After the samples with not-enough remaining cells, HPV genotype failure, and unqualified DNA were further excluded, 875 samples were finally enrolled into the study, include 214 HPV negative, 294 single HPV16 positive, 55 single HPV18 positive, 155 single HPV52 positive, and 157 single HPV58 positive. All the samples had the histological diagnosis, and the diagnostic criteria were followed by the American Society for Colposcopy and Cervical Pathology (ASCCP) 2006 guidelines [23]. Sample collection for the study was approved by the Ethics Committee of the Hospital.

### HPV test and genotyping

The HPV test was performed by the Hybrid Capture 2 (HC2) test (Qiagen Digene) or Cervista test (Hologic). HC2 test is used for detecting the pool of 13 high-risk HPV genotypes including HPV 16, 18, 31, 33, 35, 39, 45, 51, 52, 56, 58, 59 and 68. Cervista test is used for detecting 14 genotypes (above 13 types plus 66), with a report of A5/A6(51, 56, 66), A7(18, 39, 45, 59, 68), and/or A9 (16, 31, 33, 35, 52, 58) positive or negative. Hybribio Rapid GenoArray test kit (GA) is used for HPV genotyping, including 6 low-risk types (6, 11, 42, 43, 44 and CP8304), 15 high-risk types (16, 18, 31, 33, 35, 39, 45, 51, 52, 56, 58, 59, 68, 66 and 53). All the tests were performed according to the manufacturer's protocol [24–26].

### SNP selection and genotyping

The Haploview software 4.2 (Mark Daly' s lab of Broad Institute, Cambridge, MA, Britain) was used to analyze the tagSNPs and haplotype block based on the CHB (Chinese Han Beijing) population data of HapMap (HapMap Data Rel 27 PhaseII +III, Feb09, on NCBI B36 assembly, dbSNP b126 (International HapMap Project), a total of 96 SNPs in 8 HPV receptor and associated genes (*EGFR, PPIB, HSPG2, FGFR2, FURIN, ITGA6, TSPAN1* and *SDC2*) were genotyped. Validated tagSNPs were selected with a MAF > 5% in the HapMap Asia population. SNPs that satisfied the following criteria were considered for detection: 1) tagSNPs were preferentially selected, 2) those SNPs were previously reported to be frequent in Chinese population (http://www.ncbi.nlm.nih.gov/snp). The total genomic DNA was extracted from the cervical exfoliated cells using KoningTM Mutisource Genomic DNA Extration Kit-Mini and PureLink® Genomic DNA Kits (invitrogen). The DNA concentration was detected, agarose gel eletrophoresis was run and the final concentration was quantified to 50 ng/μl. All the SNPs were genotyped by Illumina BeadXpress VeraCode platform (USA), according to the manufacturer's protocol.

### Statistical analysis

All statistical analyses were performed using PLINK version 1.07 [27]. All p values in this study were two-sided by CHISQ and fisher test [28]. A p<0.05 was considered as the threshold for statistical significance. Allele frequencies, genotype frequencies and haplotypes frequencies for each SNP of all the subjects were compared using the CHISQ and fisher test. ORs and 95% CIs were calculated by unconditional logistic regression analyses adjusted for age [29]. Genotypic frequencies in control subjects for each SNP were tested for departure from HWE using an exact test. Each HPV16/18/52/58 group was divided into two subgroups according to the pathological grade(≥HSIL and ≤LSIL) for analyzing the correlation between SNPs and lesion progression. Population was not stratified because all participants' ethnicity was Han Chinese.

## SUPPLEMENTARY TABLES




