# Supplementary material for: Variants in human papillomavirus receptor and associated genes are associated with type-specific HPV infection and lesion progression of the cervix
Source: Oncotarget. 2016 May 20;7(26):40135–47. doi: 10.18632/oncotarget.9510 (PMC5129998; doi:10.18632/oncotarget.9510)
Supplement: Supplementary file 1 [file oncotarget-07-40135-s001.pdf]

Variants in human papillomavirus receptor and associated genes are associated with type-specific HPV infection and lesion progression of the cervix

SUPPLEMENTARY TABLES

Supplementary Table S1: The significant different SNP sites in target genes between single HPV16/18/52/58 positive and HPV negative in all the samples

vs HPV negative	SNP_ID	Gene	chr	position	A1	A2	HWE p value in control	OR(95%CI)	P value
HPV16	rs2651465	*SDC2*	8	97551348	T	A	0.0197	0.7154 (0.5487-0.9328)	0.01449
	rs4947972	*EGFR*	7	55161043	G	C	0.4817	1.544 (1.06-2.25)	0.02629
	rs2515127	*SDC2*	8	97519572	G	A	0.4907	1.409 (1.032-1.924)	0.03553
HPV18	rs2575712	*SDC2*	8	97576436	A	C	1	0.4829 (0.3101-0.7519)	0.001118
	rs3767137	*HSPG2*	1	22160723	A	G	1	0.3469 (0.1621-0.7424)	0.00345
	rs2575735	*SDC2*	8	97534651	A	G	1	1.725 (1.069-2.782)	0.03549
	rs10890384	*TSPAN1*	1	46649046	A	G	0.3481	0.4111 (0.1719-0.9831)	0.03999
	rs2575738	*SDC2*	8	97530402	A	G	0.696	1.639 (1.026-2.62)	0.04269
	rs6658920	*HSPG2*	1	22257739	A	G	1	0.435 (0.1927-0.9822)	0.04755
HPV52	rs2589205	*SDC2*	8	97547792	A	G	0.01783	1.402 (1.044-1.884)	0.02931
	rs6680566	*HSPG2*	1	22229090	G	A	0.477	0.7112 (0.5229-0.9674)	0.0302
	rs2253557	*PPIB*	15	64448365	G	A	1	0.4602(0.2208-0.9591)	0.04421
HPV58	rs11770506	*EGFR*	7	55090379	A	G	0.2799	1.466 (1.082-1.985)	0.01617
	rs17514846	*FURIN*	15	91416550	A	C	0.4297	0.5896 (0.3725-0.9332)	0.02622
	rs4947972	*EGFR*	7	55161043	G	C	0.4817	1.629 (1.065-2.493)	0.02831
	rs2575712	*SDC2*	8	97576436	A	C	1	0.7214 (0.537-0.9692)	0.03558

A1: Minor allele name
A2: Major allele name
OR: Estimated odds ratio (for A1, A2 is reference)
HWE: Hardy–Weinberg equilibrium

Supplementary Table S2: The significant different genotypes in target genes between single HPV16/18/52/ 58 positive and HPV negative in all the samples

See Supplementary File 1

Supplementary Table S3: The significant different haplotypes in target genes between single HPV16/18/52/ 58 positive and HPV negative in all the samples

HPV genotype vs HPV negative	Block	GENE	Haplotype	Freq (case)	Freq (control)	χ^2	P. Chi Square	OR(95%CI)	P fisher
HPV18	1	*HSPG2*	GGAGA	0.057	0.16	7.612	0.0058	0.314648 (0.108379-0.75082)	0.004508
HPV52	2	*HSPG2*	GGG	0.326	0.403	4.421	0.0355	0.718551 (0.521252-0.987964)	0.036331
	2	*HSPG2*	GGA	0.295	0.228	4.305	0.038	1.422876 (1.003471-2.017511)	0.039358
HPV58	4	*EGFR*	GG	0.578	0.667	6.133	0.0133	0.682577 (0.498071-0.934898)	0.01617
	6	*FURIN*	AA	0.097	0.152	4.724	0.0297	0.611813 (0.37181-0.98834)	0.043411

Supplementary Table S4: The different genotypes of individual SNPs in each analyzed genes in HPV negative samples

See Supplementary File 1
