# Supplementary material for: Variants in human papillomavirus receptor and associated genes are associated with type-specific HPV infection and lesion progression of the cervix
Source: Oncotarget. 2016 May 20;7(26):40135–47. doi: 10.18632/oncotarget.9510 (PMC5129998; doi:10.18632/oncotarget.9510)
Supplement: Supplementary file 2 [file oncotarget-07-40135-s002.doc]

**Table S2. The significant different genotypes in target genes between single HPV16/18/52/ 58 positive and HPV negative in all the samples**

| **vs HPV negative** | **SNP Number/Gene** | **Genotype** | **No.(frequency) in case** | **No.(frequency) in control** | **OR(95%CI)** | **P fisher** |
| --- | --- | --- | --- | --- | --- | --- |
| **HPV16** | **rs2651465**  ***SDC2*** | **TT**  **TA**  **AA** | **21(7.3%)**  **131(45.8%)**  **134(46.9%)** | **22(10.4%)**  **116(54.7%)**  **74(34.9%)** | **0.528533(0.257437-1.081239)**  **0.624314(0.419429-0.926093)**  **ref** | **0.060147**  **0.016993**  **ref** |
|  | **rs4947972**  ***EGFR*** | **GG**  **GC**  **CC** | **6(2.1%)**  **80(28.1%)**  **199(69.8%)** | **1(0.5%)**  **45(21.2%)**  **166(78.3%)** | **4.987915(0.596045-231.337)**  **1.481781(0.956747-2.314115)**  **ref** | **0.135195**  **0.075175**  **ref** |
|  | **rs2515127**  ***SDC2*** | **GG**  **GA**  **AA** | **14(4.9%)**  **110(38.6%)**  **161(56.5%)** | **5(2.4%)**  **68(32.2%)**  **138(65.4%)** | **2.393781(0.789491-8.711357)**  **1.385592(0.934006-2.063018)**  **ref** | **0.101776**  **0.104231**  **ref** |
| **HPV18** | **rs2575712**  ***SDC2*** | **AA**  **AC**  **CC** | **8(15.1%)**  **21(39.6%)**  **24(45.3%)** | **58(27.6%)**  **105(50.0%)**  **47(22.4%)** | **0.272678(0.096587-0.698705)**  **0.393682(0.187654-0.818321)**  **ref** | **0.004221**  **0.00791**  **ref** |
|  | **rs3767137**  ***HSPG2*** | **AA**  **AG**  **GG** | **1(1.9%)**  **6(11.3%)**  **46(86.8%)** | **7(3.3%)**  **66(31.4%)**  **137(65.2%)** | **0.426979(0.009246-3.468859)**  **0.271899(0.090299-0.682749)**  **ref** | **0.681865**  **0.002961**  **ref** |
|  | **rs2575735**  ***SDC2*** | **AA**  **AG**  **GG** | **5(9.4%)**  **22(41.5%)**  **26(49.1%)** | **8(3.8%)**  **69(32.5%)**  **135(63.7%)** | **3.21619(0.765375-12.22407)**  **1.652143(0.82732-3.281293)**  **ref** | **0.058093**  **0.134197**  **ref** |
|  | **rs10890384**  ***TSPAN1*** | **AA**  **AG**  **GG** | **0****(0.0%)**  **6(11.3%)**  **47(88.7%)** | **5(2.4%)**  **44(20.7%)**  **163(76.9%)** | **0(0-3.918867)**  **0.474097(0.155486-1.212458)**  **ref** | **0.588059**  **0.119719**  **ref** |
|  | **rs2575738**  ***SDC2*** | **AA**  **AG**  **GG** | **5(9.6%)**  **24(46.2%)**  **23(44.2%)** | **12(5.7%)**  **72(34.3%)**  **126(60.0%)** | **2.268755(0.57119-7.778542)**  **1.821477(0.91243-3.647832)**  **ref** | **0.169708**  **0.0694**  **ref** |
|  | **rs6658920**  ***HSPG2*** | **AA**  **AG**  **GG** | **0****(0.0%)**  **7(13.2%)**  **46(86.8%)** | **4(1.9%)**  **51(24.2%)**  **156(73.9%)** | **0(0-5.304223)**  **0.46667(0.167238-1.128971)**  **ref** | **0.577093**  **0.095402**  **ref** |
| **HPV52** | **rs2589205**  ***SDC2*** | **AA**  **AG**  **GG** | **34(22.2%)**  **92(60.1%)**  **27(17.7%)** | **32****(15.1%)**  **122(57.5%)**  **58(27.4%)** | **2.269532(1.114481-4.681472)**  **1.617337(0.925899-2.873748)**  **ref** | **0.019098**  **0.088611**  **ref** |
|  | **rs6680566**  ***HSPG2*** | **GG**  **GA**  **AA** | **20(13.1%)**  **60(39.2%)**  **73(47.7%)** | **32(15.1%)**  **108(50.9%)**  **72(34.0%)** | **0.617958(0.304485-1.2317)**  **0.54901(0.339422-0.884265)**  **ref** | **0.14909**  **0.011555**  **ref** |
|  | **rs2253557**  ***PPIB*** | **GG**  **GA**  **AA** | **0(0.0%)**  **10(6.5%)**  **143(93.5%)** | **1****(0.5%)**  **27(12.7%)**  **184(86.8%)** | **0(0-50.41559)**  **0.47747(0.199412-1.058436)**  **ref** | **1**  **0.054796**  **ref** |
| **HPV58** | **rs11770506**  ***EGFR*** | **AA**  **AG**  **GG** | **22(14.3%)**  **86(55.8%)**  **46(29.9%)** | **27(12.7%)**  **87(41.0%)**  **98(46.2%)** | **1.730795(0.842919-3.539593)**  **2.100947(1.297139-3.429275)**  **ref** | **0.119916**  **0.001953**  **ref** |
|  | **rs17514846**  ***FURIN*** | **AA**  **AC**  **CC** | **2(1.3%)**  **26(17.0%)**  **125(81.7%)** | **3(1.4%)**  **60(28.3%)**  **149(70.3%)** | **0.795313(0.065494-7.056763)**  **0.517462(0.29487-0.889611)**  **ref** | **1**  **0.012366**  **ref** |
|  | **rs4947972**  ***EGFR*** | **GG**  **GC**  **CC** | **2(1.3%)**  **48(31.2%)**  **104(67.5%)** | **1(0.5%)**  **45(21.2%)**  **166(78.3%)** | **3.178442(0.163568-189.2276)**  **1.700056(1.029087-2.814845)**  **ref** | **0.561923**  **0.028967**  **ref** |
|  | **rs2575712**  ***SDC2*** | **AA**  **AC**  **CC** | **24(15.6%)**  **89(57.8%)**  **41(26.6%)** | **58(27.6%)**  **105(50.0%)**  **47(22.4%)** | **0.476464(0.23888-0.935745)**  **0.971749(0.568605-1.663964)**  **ref** | **0.02684**  **1**  **ref** |

**Table S4. The different genotypes of individual SNPs in each analyzed genes in HPV negative samples**

| **SNP Number** | **Gene** | **genotype** | **No.** | **frequency** | **genotype** | **No.** | **frequency** | **genotype** | **No.** | **(frequency)** |
| --- | --- | --- | --- | --- | --- | --- | --- | --- | --- | --- |
| rs2981432 | *FGFR2* | AA | 77 | 36.15% | AG | 112 | 52.58% | GG | 24 | 11.27% |
| rs11487218 | *EGFR* | AA | 204 | 95.77% | AG | 9 | 4.23% | GG | 0 | 0.00% |
| rs6743724 | *ITGA6* | AA | 148 | 69.48% | AC | 59 | 27.70% | CC | 6 | 2.82% |
| rs3750817 | *FGFR2* | AA | 59 | 27.70% | AG | 106 | 49.77% | GG | 48 | 22.54% |
| rs9426785 | *HSPG2* | AA | 77 | 36.15% | AG | 105 | 49.30% | GG | 31 | 14.55% |
| rs4654771 | *HSPG2* | AA | 40 | 18.96% | AG | 100 | 47.39% | GG | 71 | 33.65% |
| rs2981430 | *FGFR2* | AA | 36 | 16.98% | AG | 111 | 52.36% | GG | 65 | 30.66% |
| rs6658920 | *HSPG2* | AA | 4 | 1.88% | AG | 52 | 24.41% | GG | 157 | 73.71% |
| rs3823585 | *EGFR* | CC | 24 | 11.27% | CG | 85 | 39.91% | GG | 104 | 48.83% |
| rs2981575 | *FGFR2* | AA | 71 | 33.33% | AG | 103 | 48.36% | GG | 39 | 18.31% |
| rs7177371 | *PPIB* | AA | 0 | 0.00% | AG | 12 | 5.63% | GG | 201 | 94.37% |
| rs2234266 | *TSPAN1* | AA | 204 | 95.77% | AG | 9 | 4.23% | GG | 0 | 0.00% |
| rs878949 | *HSPG2* | AA | 4 | 1.88% | AG | 54 | 25.35% | GG | 155 | 72.77% |
| rs10930558 | *ITGA6* | AA | 136 | 64.15% | AG | 71 | 33.49% | GG | 5 | 2.36% |
| rs12117402 | *HSPG2* | AA | 25 | 11.74% | AG | 100 | 46.95% | GG | 88 | 41.31% |
| rs2253557 | *PPIB* | AA | 193 | 90.61% | AG | 19 | 8.92% | GG | 1 | 0.47% |
| rs16894821 | *SDC2* | AA | 115 | 54.25% | AG | 85 | 40.09% | GG | 12 | 5.66% |
| rs2288336 | *FGFR2* | AA | 18 | 8.53% | AG | 100 | 47.39% | GG | 93 | 44.08% |
| rs17514846 | *FURIN* | AA | 3 | 1.42% | AC | 60 | 28.30% | CC | 149 | 70.28% |
| rs3135761 | *FGFR2* | AA | 53 | 24.88% | AG | 115 | 53.99% | GG | 45 | 21.13% |
| rs2515127 | *SDC2* | AA | 139 | 65.26% | AG | 68 | 31.92% | GG | 6 | 2.82% |
| rs5773901 | *TSPAN1* | TT | 85 | 41.26% | TC | 114 | 55.34% | CC | 7 | 3.40% |
| rs10209072 | *ITGA6* | AA | 0 | 0.00% | AG | 2 | 0.94% | GG | 210 | 99.06% |
| rs4947972 | *EGFR* | CC | 167 | 78.40% | CG | 45 | 21.13% | GG | 1 | 0.47% |
| rs2582842 | *SDC2* | AA | 81 | 38.03% | AG | 108 | 50.70% | GG | 24 | 11.27% |
| rs16860426 | *ITGA6* | AA | 11 | 5.21% | AT | 104 | 49.29% | TT | 96 | 45.50% |
| rs6759316 | *ITGA6* | AA | 22 | 10.33% | AG | 106 | 49.77% | GG | 85 | 39.91% |
| rs6697265 | *HSPG2* | CC | 61 | 28.64% | CG | 116 | 54.46% | GG | 36 | 16.90% |
| rs2254357 | *HSPG2* | CC | 29 | 13.68% | CG | 98 | 46.23% | GG | 85 | 40.09% |
| rs1047100 | *FGFR2* | AA | 1 | 0.47% | AG | 34 | 15.96% | GG | 178 | 83.57% |
| rs12034979 | *HSPG2* | AA | 0 | 0.00% | AG | 20 | 9.39% | GG | 193 | 90.61% |
| rs3767137 | *HSPG2* | AA | 7 | 3.30% | AG | 67 | 31.60% | GG | 138 | 65.09% |
| rs11199993 | *FGFR2* | CC | 12 | 5.63% | CG | 78 | 36.62% | GG | 123 | 57.75% |
| rs2737085 | *ITGA6* | CC | 210 | 98.59% | AC | 3 | 1.41% | AA | 0 | 0.00% |
| rs2556537 | *FGFR2* | AA | 62 | 29.25% | AG | 120 | 56.60% | GG | 30 | 14.15% |
| rs12718946 | *EGFR* | CC | 75 | 35.21% | CG | 105 | 49.30% | GG | 33 | 15.49% |
| rs4947982 | *EGFR* | AA | 77 | 36.15% | AG | 110 | 51.64% | GG | 26 | 12.21% |
| rs6956366 | *EGFR* | CC | 146 | 68.54% | CG | 64 | 30.05% | GG | 3 | 1.41% |
| rs4654773 | *HSPG2* | AA | 28 | 13.21% | AG | 114 | 53.77% | GG | 70 | 33.02% |
| rs7780270 | *EGFR* | AA | 184 | 86.38% | AC | 28 | 13.15% | CC | 1 | 0.47% |
| rs16860497 | *ITGA6* | AA | 106 | 49.77% | AG | 93 | 43.66% | GG | 14 | 6.57% |
| rs2936870 | *FGFR2* | AA | 32 | 15.02% | AG | 104 | 48.83% | GG | 77 | 36.15% |
| rs28384376 | *EGFR* | AA | 7 | 3.30% | AC | 67 | 31.60% | CC | 138 | 65.09% |
| rs12668175 | *EGFR* | AA | 55 | 25.82% | AC | 112 | 52.58% | CC | 46 | 21.60% |
| rs2305562 | *HSPG2* | AA | 64 | 30.05% | AG | 105 | 49.30% | GG | 44 | 20.66% |
| rs2912780 | *FGFR2* | AA | 61 | 28.91% | AG | 109 | 51.66% | GG | 41 | 19.43% |
| rs1920979 | *ITGA6* | AA | 83 | 39.15% | AG | 109 | 51.42% | GG | 20 | 9.43% |
| rs2464474 | *SDC2* | AA | 38 | 17.84% | AG | 110 | 51.64% | GG | 65 | 30.52% |
| rs2651475 | *SDC2* | AA | 22 | 57.89% | AG | 16 | 42.11% | GG | 0 | 0.00% |
| rs724236 | *SDC2* | AA | 16 | 7.51% | AT | 89 | 41.78% | TT | 108 | 50.70% |
| rs1042381 | *SDC2* | AA | 11 | 5.16% | AT | 75 | 35.21% | TT | 127 | 59.62% |
| rs2589205 | *SDC2* | AA | 32 | 15.09% | AG | 122 | 57.55% | GG | 58 | 27.36% |
| rs2981428 | *FGFR2* | AA | 38 | 18.01% | AC | 119 | 56.40% | CC | 54 | 25.59% |
| rs17337451 | *EGFR* | - | - | - | - | - | - | CC | 213 | 100% |
| rs3820082 | *TSPAN1* | AA | 11 | 5.16% | AC | 94 | 44.13% | CC | 108 | 50.70% |
| rs880938 | *SDC2* | CC | 34 | 15.96% | CG | 119 | 55.87% | GG | 60 | 28.17% |
| rs999681 | *SDC2* | AA | 68 | 31.92% | AC | 114 | 53.52% | CC | 31 | 14.55% |
| rs10890384 | *TSPAN1* | AA | 5 | 2.35% | AG | 43 | 20.19% | GG | 165 | 77.46% |
| rs3135772 | *FGFR2* | AA | 54 | 25.35% | AG | 115 | 53.99% | GG | 44 | 20.66% |
| rs2575735 | *SDC2* | AA | 8 | 3.76% | AG | 70 | 32.86% | GG | 135 | 63.38% |
| rs2981578 | *FGFR2* | AA | 63 | 29.72% | AG | 91 | 42.92% | GG | 58 | 27.36% |
| rs41286815 | *TSPAN1* | - | - | - | - | - | - | CC | 211 | 100% |
| rs6714597 | *ITGA6* | AA | 155 | 72.77% | AG | 56 | 26.29% | GG | 2 | 0.94% |
| rs1545593 | *HSPG2* | AA | 22 | 10.38% | AC | 84 | 39.62% | CC | 106 | 50.00% |
| rs2575732 | *SDC2* | CC | 2 | 0.94% | CG | 47 | 22.17% | GG | 163 | 76.89% |
| rs763317 | *EGFR* | AA | 8 | 3.77% | AG | 63 | 29.72% | GG | 141 | 66.51% |
| rs11770506 | *EGFR* | AA | 27 | 12.68% | AG | 88 | 41.31% | GG | 98 | 46.01% |
| rs1348563 | *SDC2* | AA | 51 | 23.94% | AC | 107 | 50.23% | CC | 55 | 25.82% |
| rs6698486 | *HSPG2* | AA | 87 | 41.04% | AG | 104 | 49.06% | GG | 21 | 9.91% |
| rs7518070 | *HSPG2* | AA | 11 | 5.19% | AG | 68 | 32.08% | GG | 133 | 62.74% |
| rs4654997 | *HSPG2* | AA | 0 | 0.00% | AG | 4 | 1.89% | GG | 208 | 98.11% |
| rs4947974 | *EGFR* | AA | 96 | 45.07% | AG | 97 | 45.54% | GG | 20 | 9.39% |
| rs4702 | *FURIN* | AA | 46 | 21.60% | AG | 122 | 57.28% | GG | 45 | 21.13% |
| rs13244925 | *EGFR* | AA | 23 | 10.80% | AC | 104 | 48.83% | CC | 86 | 40.38% |
| rs4904 | *PPIB* | AA | 0 | 0.00% | AC | 13 | 6.13% | CC | 199 | 93.87% |
| rs10510097 | *FGFR2* | AA | 7 | 3.29% | AG | 70 | 32.86% | GG | 136 | 63.85% |
| rs723527 | *EGFR* | AA | 0 | 0.00% | AG | 11 | 5.16% | GG | 202 | 94.84% |
| rs1047057 | *FGFR2* | AA | 31 | 14.62% | AG | 120 | 56.60% | GG | 61 | 28.77% |
| rs2981451 | *FGFR2* | AA | 19 | 8.92% | AC | 84 | 39.44% | CC | 110 | 51.64% |
| rs17664 | *ITGA6* | AA | 6 | 2.82% | AG | 52 | 24.41% | GG | 155 | 72.77% |
| rs12668421 | *EGFR* | AA | 3 | 1.42% | AT | 59 | 27.83% | TT | 150 | 70.75% |
| rs3750819 | *FGFR2* | CC | 0 | 0.00% | CG | 3 | 1.42% | CC | 209 | 98.58% |
| rs6680566 | *HSPG2* | AA | 74 | 34.74% | AG | 107 | 50.23% | GG | 32 | 15.02% |
| rs2575712 | *SDC2* | AA | 58 | 27.49% | AC | 106 | 50.24% | CC | 47 | 22.27% |
| rs1126681 | *SDC2* | AA | 28 | 13.15% | AG | 109 | 51.17% | GG | 76 | 35.68% |
| rs7536272 | *TSPAN1* | AA | 105 | 49.30% | AG | 91 | 42.72% | GG | 17 | 7.98% |
| rs10510098 | *FGFR2* | AA | 132 | 61.97% | AG | 73 | 34.27% | GG | 8 | 3.76% |
| rs2575738 | *SDC2* | AA | 12 | 5.71% | AG | 72 | 34.29% | GG | 126 | 60.00% |
| rs2582814 | *SDC2* | AA | 33 | 15.49% | AG | 121 | 56.81% | GG | 59 | 27.70% |
| rs3778866 | *EGFR* | AA | 95 | 45.02% | AC | 95 | 45.02% | CC | 21 | 9.95% |
| rs2912791 | *FGFR2* | AA | 49 | 23.00% | AG | 117 | 54.93% | GG | 47 | 22.07% |
| rs11895564 | *ITGA6* | AA | 2 | 0.94% | AG | 32 | 15.02% | GG | 179 | 84.04% |
| rs2651465 | *SDC2* | AA | 74 | 34.74% | AT | 120 | 56.34% | TT | 19 | 8.92% |
| rs13250770 | *SDC2* | AA | 186 | 87.32% | AC | 25 | 11.74% | CC | 2 | 0.94% |
| rs2981427 | *FGFR2* | AA | 64 | 30.05% | AG | 118 | 55.40% | GG | 31 | 14.55% |
| rs11977660 | *EGFR* | AA | 26 | 12.26% | AG | 91 | 42.92% | GG | 95 | 44.81% |
